# CD52/FLAG and CD52/HA Fusion Proteins as Novel Magnetic Cell Selection Markers

**DOI:** 10.3390/ijms25126353

**Published:** 2024-06-08

**Authors:** Oleg F. Kandarakov, Natalia S. Polyakova, Alexandra V. Petrovskaya, Alexandra V. Bruter, Alexander V. Belyavsky

**Affiliations:** 1 Engelhardt Institute of Molecular Biology, Russian Academy of Sciences, Vavilov Str. 32, 119991 Moscow, Russia; oleg.kandarakov@gmail.com (O.F.K.); natae05@yandex.ru (N.S.P.); alexvalpetrovskaya@gmail.com (A.V.P.); 2 Center for Precision Genome Editing and Genetic Technologies for Biomedicine, Vavilov Str. 32, 119991 Moscow, Russia; aleabruter@gmail.com; 3 Institute of Gene Biology, Russian Academy of Sciences, Vavilov Str. 34/5, 119334 Moscow, Russia

**Keywords:** HA tag, FLAG tag, LNGFR, EGFP, DsRedExpress2, magnetic selection, MACS selection

## Abstract

At present, the magnetic selection of genetically modified cells is mainly performed with surface markers naturally expressed by cells such as CD4, LNGFR (low affinity nerve growth factor receptor), and MHC class I molecule H-2Kk. The disadvantage of such markers is the possibility of their undesired and poorly predictable expression by unmodified cells before or after cell manipulation, which makes it essential to develop new surface markers that would not have such a drawback. Earlier, modified CD52 surface protein variants with embedded HA and FLAG epitope tags (CD52/FLAG and CD52/HA) were developed by the group of Dr. Mazurov for the fluorescent cell sorting of CRISPR-modified cells. In the current study, we tested whether these markers can be used for the magnetic selection of transduced cells. For this purpose, appropriate constructs were created in MigR1-based bicistronic retroviral vectors containing EGFP and DsRedExpress2 as fluorescent reporters. Cytometric analysis of the transduced NIH 3T3 cell populations after magnetic selection evaluated the efficiency of isolation and purity of the obtained populations, as well as the change in the median fluorescence intensity (MFI). The results of this study demonstrate that the surface markers CD52/FLAG and CD52/HA can be effectively used for magnetic cell selection, and their efficiencies are comparable to that of the commonly used LNGFR marker. At the same time, the significant advantage of these markers is the absence of HA and FLAG epitope sequences in cellular proteins, which rules out the spurious co-isolation of negative cells.

## 1. Introduction

Progress in experimental biology, which was additionally spurred by the advent of cell therapeutic applications, has resulted in considerable interest in the methods of ex vivo cell separation [1,2,3,4]. The choice of a cell selection strategy is based on many parameters; however, the main criteria include the effectiveness of cell selection, the purity of the obtained populations and the preservation of cell properties after selection [5]. One of the important directions in this vast field is the specific selection of genetically modified cells. In this case, markers that include various reporter and surface proteins, as well as antibiotic resistance factors, are required for cell selection [6]. Cell selection approaches depend on objectives and markers used, and range from simple antibiotic cell selection to fluorescence-activated cell sorting (FACS) and magnet-activated cell sorting (MACS). The unquestionable advantages of magnetic selection include the simplicity of the procedure and its ability to process large numbers of cells in a short time [7,8,9]. In addition, this approach makes it possible to select rare populations [10,11], as well as populations with high and low levels of surface marker expression [12]. For the magnetic selection of genetically modified cells, approaches based on the use of streptavidin-biotin systems might be used, including the biotinylation of the surface tag via co-expression in cells of biotin ligase BirA [13], the expression of streptavidin [14] or streptavidin-binding peptide on the cell surface [15]. These approaches, however, have not become widespread; instead, certain surface markers naturally expressed by cells are primarily used for the magnetic selection of modified cells, in particular CD4 [16,17] and LNGFR [10,12,18]. Commercial kits (Miltenyi Biotec) use LNGFR, CD4 and H-2Kk markers for the magnetic selection of transiently modified cell populations. The disadvantage of such natural markers is that they can be expressed by unmodified cells both before and (as is especially dangerous) after manipulations with cells, which can lead to the co-isolation of negative cells, the reduced quality of selection and the incorrect interpretation of the results. For example, LNGFR, in addition to cells of neural origin, is expressed in cells originating from all three germ layers [19], including neural crest cells [20]. LNGFR is also expressed by cells holding great promise for cell therapy, namely, mesenchymal stem cells (MSCs) from various tissues [21,22], including subpopulations of MSCs with angiogenic potential [23,24]. It is important to emphasize that while MSCs originally express LNGFR, they gradually lose it during cultivation [25]. On the other hand, MSCs may re-express LNGFR during adipose differentiation [26]. Such a highly complex and dynamic expression pattern cannot a priori be excluded for other naturally expressed marker proteins.

These considerations make it highly important to develop new surface markers that would not have such a disadvantage. In this regard, a promising strategy is the use of epitope tags converted to be associated with cell surface proteins. Amongst the most popular epitope tags are the FLAG and HA tags, which are often used to purify epitope-labeled recombinant proteins [27,28] in studies related to the structure and subcellular localization of proteins [29], as well as in the genetic modification of cells [30]. The potential attractiveness of FLAG and HA tags for the magnetic selection of transduced cells is, firstly, due to their small size, which makes it possible to embed them in surface proteins without changing the properties of the latter, thereby providing the access of tags to antibodies associated with magnetic particles. Secondly, the epitopes of HA and FLAG tags differ from the epitopes of animal cell proteins, which excludes the selection of untransduced cells. Finally, highly specific monoclonal antibodies to HA and FLAG tags are commercially available.

Earlier, two modifications of the glycosylphosphatidylinositol (GPI)-anchored surface marker CD52, produced by means of embedding HA and FLAG tags within the CD52 sequence, were developed for the effective FACS selection of CRISPR-modified cells [31]. Since both the reagents and the conditions and requirements for fluorescent and magnetic sorting differ significantly, and the applicability of the developed CD52/HA and CD52/FLAG markers for magnetic selection was not a priori guaranteed, we showed in this study the possibility of using these two markers for the effective magnetic selection of transduced cells, and also compared their work with that of the LNGFR selection marker.

## 2. Results

### 2.1. Retroviral Constructs

**Figure 1:**
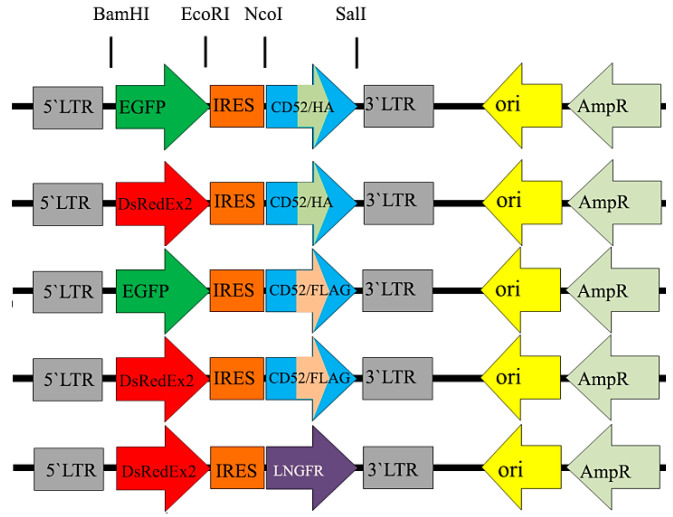
Bicistronic expression constructs used in this study. The constructs were prepared on the basis of retroviral MigR1 vector as described in the Materials and Methods.

To adequately analyze the applicability of new CD52-based markers for magnetic cell selection and compare them with the common LNGFR marker, bicistronic retroviral constructs with fluorescent protein reporters were created, which were analogous to those used by us previously [12], in which EGFP or DsRedExpress2 reporters were in the first position and the LNGFR marker was in the second position of the bicistronic unit. It is important to note that in the aforementioned work, a strict correlation was shown between the expression of reporter proteins and the LNGFR marker by transduced cells. In the retroviral vectors used in the current study, the LNGFR marker in the second position was replaced by CD52/FLAG or CD52/HA markers, while the fluorescent reporters remained in the first position. Figure 1 shows the retroviral vectors that were used for the subsequent transduction of NIH 3T3 cells.

### 2.2. Transduction of NIH 3T3 Cells

**Figure 2:**
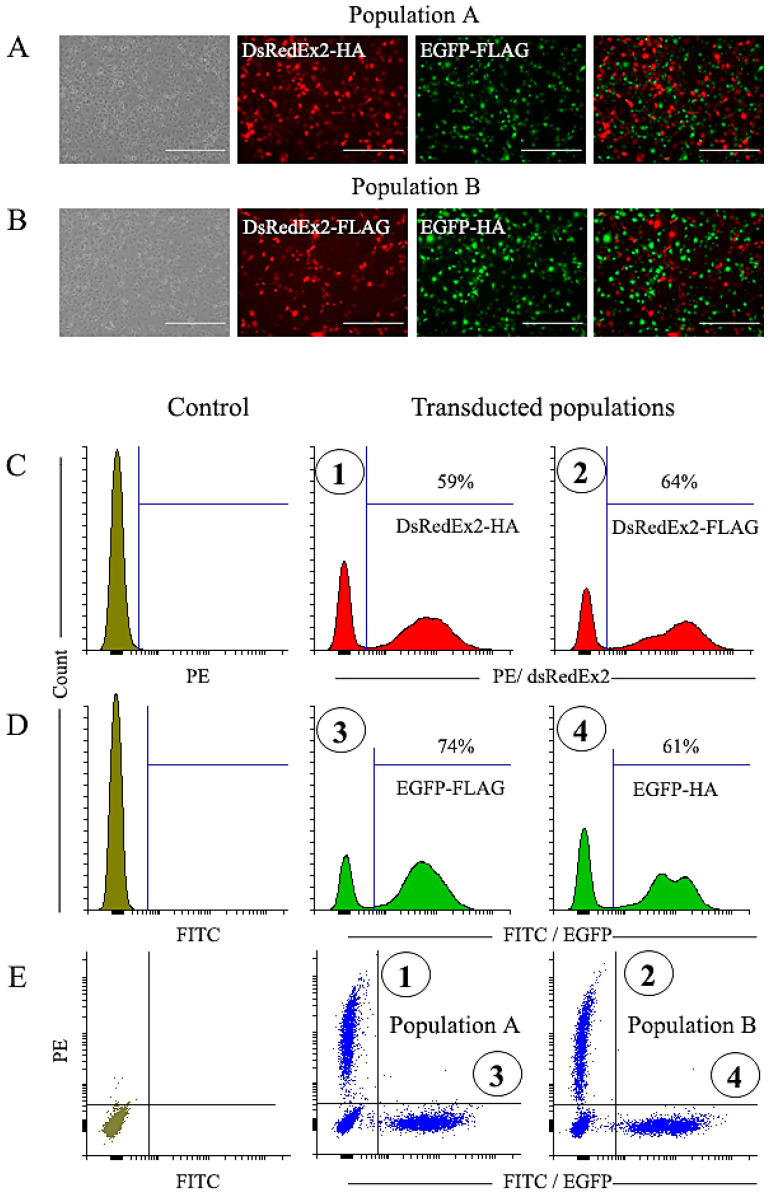
The transduction of NIH 3T3 cells with the bicistronic constructs expressing DsRedExpress2 or EGFP reporters, and the creation of mixed cell populations. (**A**,**B**) Qualitative assessment of mixed populations of DsRedEx2-HA and EGFP-FLAG cells (panel **A**) and DsRedEx2-FLAG and EGFP-HA (panel **B**) by fluorescence photomicrography. They represent the populations A and B, respectively, which are depicted quantitatively in panel E. The original cell populations were obtained by the transduction of NIH 3T3 cells with the bicistronic constructs expressing DsRedExpress2 (panel **C**) or EGFP (panel **D**) reporters. Cell populations numbered 1–4 (circled) were used to create mixed populations (panel **E**) that were subsequently subjected to magnetic selection as depicted in Figure 3. Control–untransduced NIH 3T3 cells. The bar size is 400 µm.

As a result of the transduction of NIH 3T3 cells by a set of vectors shown in Figure 1, five cell populations were obtained. Cytometric analysis showed that the fraction of EGFP-positive and DsRedExp2-positive cells among the variants varied, but in all cases was more than 50% (Figure 2). For a more unambiguous interpretation of the selection results and to ensure the possibility of selection by two markers in one cell sample, we prepared two mixed populations, A and B, combining cells with different tags and reporter proteins (Figure 2). For mixed population A, DsRedEx2-HA and EGFP-FLAG variants were combined (marked in Figure 2 with numbers 1 and 3, respectively). For mixed population B, DsRedEx2-FLAG and EGFP-HA variants were used (marked in Figure 2 with numbers 2 and 4, respectively).

### 2.3. Magnetic Selection of NIH 3T3 Transduced Cells

#### 2.3.1. Magnetic Selection of FLAG+ and HA+ Cells

For an adequate comparison of the effectiveness of various surface markers used in selection, it is important not only to use expression constructs with similar structures, but also to employ cell populations with a similar degree of transduction, as well as use identical conditions for magnetic selection, with critical reagents procured from the same manufacturer. In addition, the concentration of antibodies, the number of cells to be selected, and the proportion of reporter protein-positive cells in populations before selection in all experiments should be similar. Finally, for the most correct comparison, the conditions of cell selection should preferably be unsaturated with intermediate concentrations of the antibodies employed, thus resulting in only a fraction, but not all, of the positive cells of the original population being selected.

**Figure 3:**
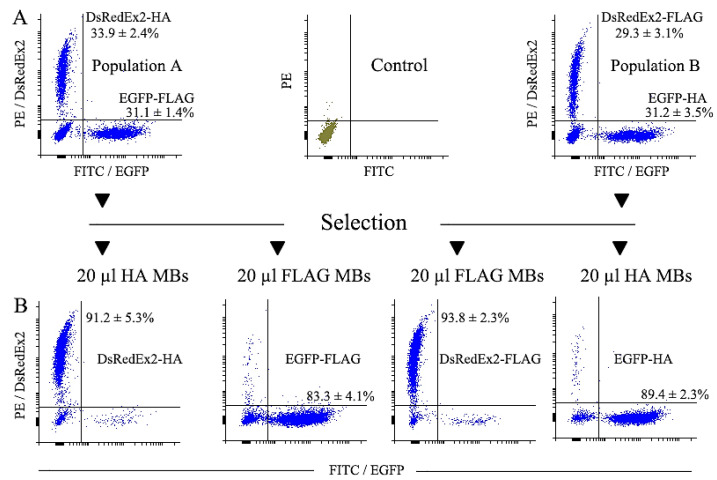
Magnetic selection of two mixed populations of transduced NIH 3T3 cells (see Figure 2). (**A**,**B**) Histograms of cells before and after magnetic selection with 20 μL of MBs. Control–untransduced NIH 3T3 cells.

**Figure 4:**
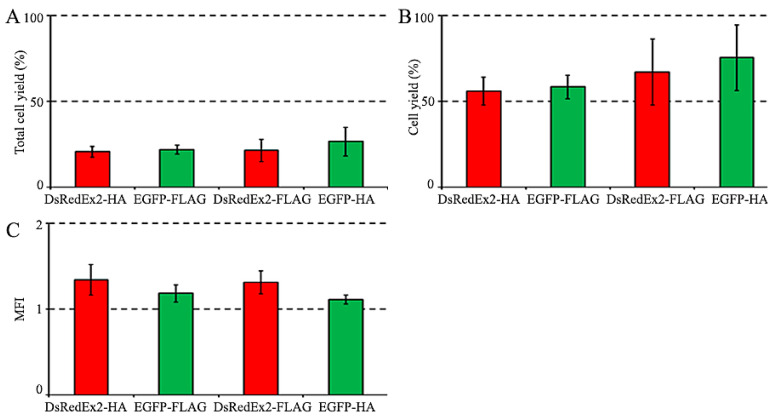
Magnetic selection of two mixed populations of transduced NIH 3T3 cells (see Figure 3). (**A**) The yield of total cells after selection (defined as the ratio of cell numbers in selected and starting populations). (**B**) The yield of EGFP+ or DsRedExp2+ cells after selection (defined as the ratio of EGFP+ or DsRedExp2+ cell numbers in selected and starting populations). (**C**) MFI (vertical axis) depicts the ratio of MFI values after and before selection. The number of cells before selection (1 × 10^6^) was taken as 100%.

At the first stage of comparison, we performed magnetic selection of the transduced cells by FLAG and HA tags. The selection results were evaluated by changing the proportions of EGFP- and DsRedExp2-positive cells in the population after magnetic selection. The cytometry of cells of mixed populations A and B before selection showed similar amounts of EGFP and DsRedEx2 cells, and their proportions in mixed populations were about 30% (Figure 3A). Each mixed population was divided into two equal parts, one of which was used for magnetic cell selection with FLAG tag, and the other by HA tag. Magnetic cell selection was performed with 20 µL of anti-FLAG MBs or 20 µL of anti-HA MBs. The cytometry of cells before and after magnetic selection is shown in Figure 3. The percentage of cells selected from the total population differed slightly between the variants and ranged from 20.5 ± 3.2% for DsRedEx2-HA to 26.5 ± 8.4% for EGFP-HA (Figure 4A). The purity of selected cells was about 90% (ranging from 89.4% to 93.8%), although for the EGFP-FLAG variant it was somewhat lower (83.4%) (Figure 3B). The efficiency of magnetic selection (calculated as the fraction of cells selected from the marker-positive population) ranged from 55.9 ± 8.2% for DsRedEx2-HA to 74.5 ± 19.2% for the EGFP-HA variant (Figure 4B). Since the efficiency of selection in all cases was essentially lower than 100%, the conditions of magnetic cell selection were adequate for the appropriate comparison of selection parameters between variants. The MFI (median fluorescent intensity, reflects average fluorescence level of population) value for the magnetic selection of variants was slightly higher for DsRedEx2 reporter than for EGFP, regardless of the tag by which the selection was carried out (Figure 4). In general, no significant differences between CD52/FLAG and CD52/HA markers were observed during magnetic selection.

#### 2.3.2. Magnetic Selection of LNGFR+ Cells

**Figure 5:**
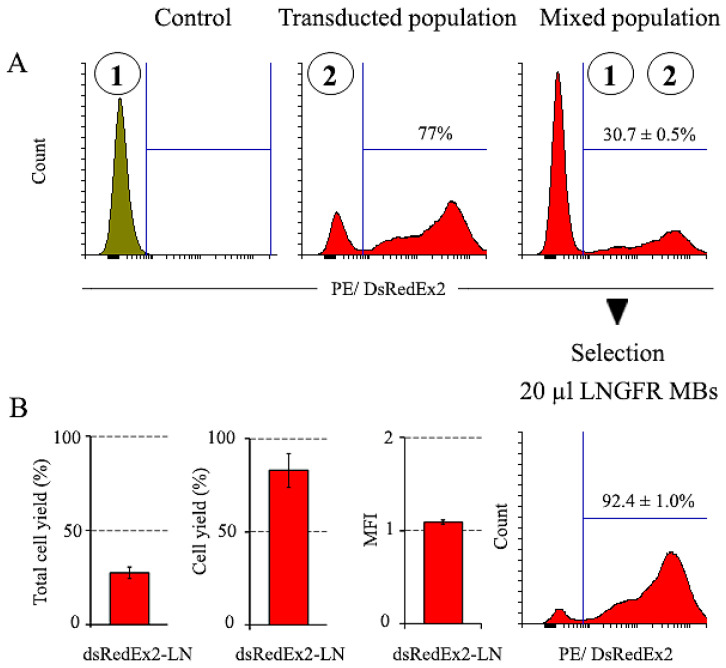
Magnetic selection of a mixed population of NIH 3T3 cells expressing LNGFR surface marker. (**A**) Cell populations numbered 1–2 (circled) were used to create mixed population; selection was performed using 20 μL of anti-LNGFR MBs. (**B**) The yield of total cells and DsRedExp2+ cells after selection, and the change in MFI expressed as the ratio of values of MFI after to MFI before selection. The number of cells before selection (1 × 10^6^) was taken as 100%. Control–untransduced NIH 3T3 cells.

At the next stage, we compared the results of the magnetic selection of cells expressing CD52/FLAG and CD52/HA markers with those of cells expressing the LNGFR marker. For this, the magnetic selection of LNGFR cells was carried out under conditions similar to those used for the selection of CD52/FLAG and CD52/HA. After the transduction of NIH 3T3 cells by the pMigLNR2-DsRex vector, the fraction of DsRedEx2-positive cells in the population constituted more than 75% (Figure 5). To reduce the proportion of positive DsRedEx2 cells in the population to 30%, the transduced and untransduced populations of NIH 3T3 cells were mixed. The cytometry of the initial and mixed cell populations is shown in Figure 5. The magnetic selection of the mixed population was performed with 20 µL of anti-LNGFR MBs (Figure 5). As a result of selection, the proportion of DsRedExp2+ cells increased from 30.7 ± 1.5% to 92.4 ± 1.0%, while the proportion of selected DsRedEx2+ cells from the mixed population was close to 83% (Figure 5). The MFI value changed slightly during selection. The results of the magnetic selection of cell populations with different surface markers are shown in Table 1.

## 3. Discussion

Various methods for selecting genetically modified cells have specific advantages and drawbacks. For example, FACS (fluorescent-activated cell sorting) has very high resolving power and may separate cells according to many markers and a number of parameters, but requires very expensive equipment, is able to process relatively small numbers of cells and may harm mid- and large size cells. On the other hand, the method of magnetic cell selection [3,7,32] has relatively low resolving power but stands out for its simplicity and speed, low negative impact on cells and the ability to work with large, including clinically relevant, populations. In the present work, we investigated the applicability of fused protein surface markers CD52/HA and CD52/FLAG, used initially for the FACS isolation of CRISPR-edited cells, in the magnetic selection of genetically modified cells. Special attention was paid to the adequate comparison of the selection efficiency attained with these markers. In addition, to analyze the work of new markers in the context of more traditional approaches, a comparison was made with the often-used magnetic selection marker LNGFR. All these comparisons were carried out using bicistronic retroviral vectors identical in structure and under identical conditions of cell selection.

The results of the study show that CD52/HA and CD52/FLAG surface markers can be effectively used in magnetic cell selection. Of note, although the FLAG- and HA-specific magnetic beads used in this study are intended by the manufacturer to be used for the isolation of tag-fused proteins, they apparently work adequately with cells as well. Since FLAG and HA tags are absent in vertebrate proteins, which minimizes the chances of the accidental co-isolation of unwanted cells, CD52/HA and CD52/FLAG markers are a promising alternative to natural markers commonly used in the magnetic selection of genetically modified cells. In addition, a significant advantage of the markers we used is their minimum size, less than 200 base pairs, which is several times smaller than the size of other markers, and is of great importance in the case of using expression vectors with limited packaging potential, such as AAV.

It is worth noting that the efficiency of CD52/HA and CD52/FLAG, although comparable to the efficiency of LNGFR, was slightly lower than the latter. A possible reason for this may be the possibility of a less-than-optimal configuration of HA and FLAG tags after insertion into CD52, which may lead to a certain decrease in their availability for the corresponding antibodies. As an alternative explanation, it can also be assumed that the affinity of antibodies in the case of LNGFR is slightly higher than in the case of HA and FLAG tags. It would be interesting in the future to investigate the effect of the antibody affinity on the effectiveness of magnetic selection. It should also be noted as an interesting fact that the purity of positive cell selection for cells with DsRedExp2 was slightly higher for both tags than for cells with EGFP. As we showed previously [12], a higher level of surface marker expression gives a higher purity of the population after magnetic selection. In this regard, it can be assumed that when DsRedExp2 is expressed by cells, a higher expression of CD52/HA and CD52/FLAG markers is achieved, which, in turn, would lead to an increase in the degree of purity of the isolated populations. It should be noted that no complete elimination of negative cells during magnetic selection was achieved, and the purity of the selected cells did not reach 100%; however, this result is generally consistent with the data of previously published studies [9,33].

It should also be mentioned that the FLAG tag embedded in tetraspanin CD63 has recently been used for the magnetic enrichment of cells [34]. According to the results of this work, however, it remains unclear how successful the use of such a marker was in terms of the yield of selected cells and the purity of the isolated population, although, by indirect indications, it was moderate. In our work, these issues were properly addressed, including the comparison with the traditional magnetic selection marker LNGFR. It is also worth mentioning the several-fold reduced size of the markers we used compared to that of CD63, which might be a significant advantage in some applications.

Finally, we would like to issue a cautionary note for the potential users of our technique. Like any other method, it has its own drawbacks and limitations. We envisage three major potential groups of causes of the inadequate functioning of this approach. First, the expression of the selection marker may be too low in the particular cell type to ensure adequate positive cell isolation. This, however, does not relate to the efficiency of the selection marker itself, and must be addressed by using promoters and/or enhancers that work more efficiently in a given cell system. Second, the selection marker may be somehow masked or shielded by other cell surface molecules from the proper interaction of FLAG or HA tags with antibodies. This is a potential jeopardy, but to our best knowledge, no known CD52 interaction partners have been demonstrated so far to possess the masking function in question. Third, the overexpression of the CD52 marker (just as any other selection marker) may result in certain disturbances of selected cell signaling pathways, which, in the worst case scenario, may result in the loss of the envisaged effect or the appearance of an undesired effects. Although the CD52 is a GPI-anchored protein and thus does not possess intracellular signaling modules, the mentioned disturbance remains a possibility, which should be addressed by a careful study of transcriptome or proteome changes in cells following marker expression.

## 4. Materials and Methods

### 4.1. Cell Cultures

NIH 3T3 cells were obtained from the Cell Culture Collection of the Institute of Cytology (Russian Academy of Sciences, Saint Petersburg, Russia). HEK 293 cells were kindly provided by Dr. V. Prassolov (Engelhardt Institute of Molecular Biology, Russian Academy of Science, Moscow, Russia). Both cell lines were cultured at 37 °C and 5% CO_2_ in a DMEM medium (HyClone Laboratories, South Logan, UT, USA) containing 4.5 g/L glucose, 10% fetal bovine serum (FBS) (HyClone), 2 mM L-glutamine (Gibco, Gaithersburg, MD, USA), 100 units/mL penicillin and 100 µg/mL streptomycin (Gibco). Cells were detached from the culture vessels by TrypLE Express (Gibco) treatment for 2–5 min at 37 °C.

### 4.2. Retroviral Constructs

The two variants of the human CD52 open reading frame (ORF) containing embedded FLAG or HA epitope tags were amplified from the plasmids pUCHR-mClover-sAID_CD5Flag2 and pUCHR-mClover-sAID_CD5HA2 (kindly provided by Dr. Mazurov), and cloned into the pTZ57R/T plasmid vector, followed by insert sequence verification. The hCD52/HA and hCD52/FLAG ORFs were excised by BspH I/Sal I digestion and re-cloned into the 2nd position of the bicistronic MigR1ad1 retroviral expression vector (described in [12]) via Nco I/Sal I sites to obtain MigRCD52HA and MigRCD52Fl vectors.

The final constructs used in this study, namely, constructs pMigRCD52H-EGFP and pMigRCD52Fl-EGFP, were prepared by re-cloning the EGFP ORF from the pMigLNR2-EGFP construct [12] into the 1st position of the MigRCD52HA and MigRCD52Fl vectors, respectively, via BamH I/EcoR I sites, while constructs pMigRCD52H-DsRex and pMigRCD52Fl-DsRex were similarly prepared by the transfer of the DsRedExpress2 ORF from the pMigLNR2-DsRex construct via BamH I/EcoR I sites of the above vectors.

The preparation of a bicistronic retroviral construct with DsRedExpress2 reporter in the 1st position and hLNGFR selection marker in the 2nd position (pMigLNR2-DsRex) was described earlier [12].

### 4.3. Transduction of the NIH 3T3 Cells

The transfection of HEK 293 cells to obtain viral particles was performed in a 10 cm Petri dish using the calcium phosphate method when the cells reached 35–45% confluence. A calcium phosphate solution with 10 μg of the relevant retroviral plasmid (Figure 1) and 10 μg of pCL-Eco plasmid (Addgene#12371) was added to the culture medium. After 12 h, the medium was replaced with a fresh one, and after an additional 24 h, the viral medium was collected, centrifuged for 10 min at 400× *g* at 4 °C, and the supernatant was passed through a 0.45 μm filter (Corning, Glendale, AR, USA).

The transduction of NIH 3T3 cells was carried out in a 6-well plate, using a seeding density of 20,000 cells/cm^2^. After reaching 35–40% cell confluence, the medium was discarded and replaced with a 1:1 mixture of viral supernatant and fresh medium. Polybrene (Sigma-Aldrich, St. Louis, MO, USA) was added thereafter to a concentration of 16 µg/mL, and cells were further cultured for 72 h, followed by the replacement of virus-containing medium with a fresh one. The efficiency of cell transduction was analyzed cytometrically 3–4 days thereafter by determining the fraction of cells in the sample expressing the reporter protein. As a result, transduced cell populations NIH 3T3-DsRedEx2-CD52/HA, NIH 3T3-DsRedEx2-CD52/FLAG, NIH 3T3-EGFP-CD52/HA, NIH 3T3-EGFP-CD52/FLAG and NIH 3T3-DsRedEx2-LNGFR were obtained, hereafter referred to as DsRedEx2-HA, DsRedEx2-FLAG, EGFP-HA, EGFP-FLAG, and DsRedEx2-LN, respectively. The transduced cells were further cultured in DMEM medium with the above-mentioned additives for 2–4 weeks before using for experiments. For the magnetic selection of cells with CD52/HA and CD52/FLAG markers, the cell populations transduced with construct variants differing in marker and reporter protein were combined so that the numbers of DsRedEx2 and EGFP cells in a mixed population were equal. Fluorescent photographs of mixed cell populations were taken with an automated microscope Lionheart™ FX (BioTek, Winooski, VT 05404, USA).

### 4.4. Magnetic Selection of Transduced Cells

The selection of the transduced cells was performed with equipment, consumables and reagents from Miltenyi Biotec (Bergisch Gladbach, Germany), including an OctoMACS Separator (No. 130-042-109), MS columns (No. 130-042-201), microbead-conjugated antibodies against FLAG epitope (µMACS DYKDDDDK isolation kit, No.130-101-591) and against HA epitope (µMACS HA isolation kit, No.130-091-122), as well as MACSelect anti-LNGFR Micro Beads (130-091-330). These microbead-conjugated antibodies are hereafter referred to as anti-FLAG MBs, anti-HA MBs and anti-LNGFR MBs, respectively. After the incubation of cells with MBs, magnetic selection was performed in accordance with the recommendations of the manufacturer for anti-LNGFR MBs. In short, the transduced cells were detached with TripLE Express solution (Gibco) for 5 min at 37 °C. The collected cells were centrifuged for 5 min at 150× *g* at 4 °C, and the pellet was resuspended in phosphate buffer with 1 mM EDTA and 0.5% FBS. The cells were counted using a hemocytometer, and aliquots of 0.8–1.2 × 10^6^ cells were distributed into 15 mL tubes. The cells were again centrifuged for 10 min at 150× *g*, and cell pellets were suspended in 380 µL of phosphate buffer with 2 mM EDTA and 0.5% FBS (PBE). Next, 20 µL of relevant MBs was added to the cell suspension and incubated for 15 min on ice. The volume of the cell suspension was then adjusted to 2 mL with PBE and applied in factions of 0.5 mL to MS column mounted on the OctoMACS magnet; thereafter, the column was washed with 2 mL of PBE buffer. To collect the selected cells, the column was removed from the magnet and placed in a 15 mL tube; 1 mL of PBE buffer was added to the MS column and the buffer was forced through the column with a plunger. The cells were counted using a hemocytometer. Cell viability was determined using Trypan blue.

### 4.5. Flow Cytometry

Cells before and after magnetic selection were collected as described above and analyzed on a BD LSRFortessa cell analyzer (BD Life Sciences, Franklin Lakes, NJ, USA) equipped with a 488 nm laser with 530 ± 15 nm emission filter and a 561 nm laser with a 586 ± 7.5 nm emission filter, which were used to detect EGFP and DsRedExpress2 fluorescence. EGFP and DsRedExpress2 emissions were compensated using BD FACSDiva software version 8.0.1 (build 2014 07 03 11 47). Cell clusters and debris were eliminated by the sequential gating of cells for size and granularity (FSC-A/SSC-A), as well as for area and peak height (FSC-A/FSC-H). The cytometric settings were kept unchanged during the measurement of all samples. The data collected for each sample were from at least 10,000 events. Cytometric data were processed using the Flowing Software version 2.5.1 1 (https://bioscience.fi/services/cell-imaging/flowing-software (accessed on 4 November 2013) on the basis of which the necessary calculations of the parameters of the transduced populations were carried out.

### 4.6. Statistical Analysis

All experiments were performed at least three times, and the data are presented as average values ± standard deviation.

## 5. Conclusions

The results of this work demonstrate that the surface markers CD52/FLAG and CD52/HA, along with LNGFR, can be effectively used for the magnetic selection of genetically modified cells. A comparison of the purity and efficiency of cell selection and MFI did not show significant differences between the three markers; however, the results of the selection of cells with LNGFR turned out to be slightly better than those of cells with HA and FLAG tags. At the same time, the absence of HA and FLAG epitopes in cellular proteins, along with the small sizes of the markers used, eliminates the possibility of the unintended co-isolation of negative cells, and represents a significant advantage over LNGFR and other natural proteins used as selective markers in magnetic selection.

## Figures and Tables

**Table 1:** Characteristics of populations with different surface markers after magnetic selection.

Scheme	Total Yield (%)	Cell Yield (%)	Purity (%)	MFI Change *
DsRedExp2-HA	20.5 ± 3.2	55.9 ± 8.2	91.2 ± 5.3	1.34 ± 0.18
EGFP-FLAG	21.7 ± 2.5	58.3 ± 6.9	83.3 ± 4.1	1.18 ± 0.10
DsRedExp2-FLAG	21.2 ± 6.6	67.0 ± 19.3	93.8 ± 2.3	1.31 ± 0.14
EGFP-HA	26.5 ± 8.4	74.5 ± 19.2	89.4 ± 2.3	1.11 ± 0.05
DsRedExp2-LNGFR	27.5 ± 3.0	82.9 ± 9.0	92.4 ± 1.0	1.09 ± 0.02

* MFI expressed as the ratio of values of MFI after to MFI before selection.

## Data Availability

Original data are available upon request.

## References

[1] de Wynter E.A., Coutinho L.H., Pei X., Marsh J.C., Hows J., Luft T., Testa N.G. (1995). Comparison of Purity and Enrichment of CD34^+^ Cells From Bone Marrow, Umbilical Cord and Peripheral Blood (Primed for Apheresis) Using Five Separation Systems. Stem Cells.

[2] Dainiak M.B., Kumar A., Galaev I.Y., Mattiasson B. (2007). Methods in cell separations. Adv. Biochem. Eng. Biotechnol..

[3] Plouffe B.D., Murthy S.K., Lewis L.H. (2015). Fundamentals and Application of Magnetic Particles in Cell Isolation and Enrichment: A review. Rep. Prog. Phys..

[4] Frenea-Robin M., Marchalot J. (2022). Basic principles and recent advances in magnetic cell separation. Magnetochemistry.

[5] Amos P.J., Bozkulak E.C., Qyang Y. (2012). Methods of Cell Purification: A Critical Juncture for Laboratory Research and Translational Science. Cells Tissues Organs.

[6] Mortensen R.M., Kingston R.E. (2009). Selection of transfected mammalian cells. Curr. Protoc. Mol. Biol..

[7] Schmitz B., Radbruch A., Kümmel T., Wickenhauser C., Korb H., Hansmann M., Thiele J., Fischer R. (1994). Magnetic activated cell sorting (MACS)—A new immunomagnetic method for megakaryocytic cell isolation: Comparison of different separation techniques. Eur. J. Haematol..

[8] Sutermaster B.A., Darling E.M. (2019). Considerations for high-yield, high-throughput cell enrichment: Fluorescence versus magnetic sorting. Sci. Rep..

[9] Pan J., Wan J. (2020). Methodological comparison of FACS and MACS isolation of enriched microglia and astrocytes from mouse brain. J. Immunol. Methods.

[10] Lee M.Y., Lufkin T. (2012). Development of the “Three-step MACS”: A novel strategy for isolating rare cell populations in the absence of known cell surface markers from complex animal tissue. J. Biomol. Technol..

[11] Wang Z., Wang H., Lin S., Ahmed S., Angers S., Sargent E.H., Kelley S.O. (2022). Nanoparticle Amplification Labeling for High-Performance Magnetic Cell Sorting. Nano Lett..

[12] Polyakova N., Kandarakov O., Belyavsky A. (2023). Selection of cell populations with high or low surface marker expression using magnetic sorting. Cells.

[13] Han H., Liu Q., He W., Ong K., Liu X., Gao B. (2011). An efficient vector system to modify cells genetically. PLoS ONE.

[14] Gotoh H., Matsumoto Y. (2007). Cell-surface streptavidin fusion protein for rapid selection of transfected mammalian cells. Gene.

[15] Matheson N.J., Peden A.A., Lehner P.J. (2014). Antibody-free magnetic cell sorting of genetically modified primary human CD4^+^ T cells by one-step streptavidin affinity purification. PLoS ONE.

[16] Gaines P., Wojchowski D.M. (1999). pIRES-CD4t, a dicistronic expression vector for MACS-or FACS-based selection of transfected cells. Biotechniques.

[17] David R., Groebner M., Franz W.M. (2005). Magnetic cell sorting purification of differentiated embryonic stem cells stably expressing truncated human CD4 as surface marker. Stem Cells.

[18] Kaufman W.L., Kocman I., Agrawal V., Rahn H.-P., Besser D., Gossen M. (2008). Homogeneity and persistence of transgene expression by omitting antibiotic selection in cell line isolation. Nucleic Acids Res..

[19] Thomson T.M., Rettig W.J., Chesa P.G., Green S.H., Mena A.C., Old L.J. (1988). Expression of human nerve growth factor receptor on cells derived from all three germ layers. Exp. Cell Res..

[20] Zhang J., Duan X., Zhang H., Deng Z., Zhou Z., Wen N., Smith A.J., Zhao W., Jin Y. (2006). Isolation of neural crest-derived stem cells from rat embryonic mandibular processes. Biol. Cell.

[21] Quirici N., Soligo D., Bossolasco P., Servida F., Lumini C., Deliliers G.L. (2002). Isolation of bone marrow mesenchymal stem cells by anti-nerve growth factor receptor antibodies. Exp. Hematol..

[22] Barilani M., Banfi F., Sironi S., Ragni E., Guillaumin S., Polveraccio F., Rosso L., Moro M., Astori G., Pozzobon M. (2018). Low-affinity Nerve Growth Factor Receptor (CD271) Heterogeneous Expression in Adult and Fetal Mesenchymal Stromal Cells. Sci. Rep..

[23] Sasse S., Skorska A., Lux C.A., Steinhoff G., David R., Gaebel R. (2020). Angiogenic Potential of Bone Marrow Derived CD133^+^ and CD271^+^ Intramyocardial Stem Cell Trans-Plantation Post MI. Cells.

[24] Smith R.J.P., Faroni A., Barrow J.R., Soul J., Reid A.J. (2021). The angiogenic potential of CD271^+^ human adipose tissue-derived mesenchymal stem cells. Stem Cell Res. Ther..

[25] Schäck L.M., Noack S., Weist R., Jagodzinski M., Krettek C., Buettner M., Hoffmann A. (2013). Analysis of surface protein expression in human bone marrow stromal cells: New aspects of culture-induced changes, inter-donor differences and intracellular expression. Stem Cells Dev..

[26] Walmsley G.G., Atashroo D.A., Maan Z.N., Hu M.S., Zielins E.R., Tsai J.M., Duscher D., Paik K., Tevlin R., Marecic O. (2015). High-Throughput Screening of Surface Marker Expression on Undifferentiated and Differentiated Human-Derived Stromal Cells. Tissue Eng. Part A.

[27] Zhao X., Li G., Liang S. (2013). Several affinity tags commonly used in chromatographic purification. J. Anal. Methods Chem..

[28] Lafuente-González E., Guadaño-Sánchez M., Urriza-Arsuaga I., Urraca J.L. (2023). Core-Shell Magnetic Imprinted Polymers for the Recognition of FLAG-Tagpeptide. Int. J. Mol. Sci..

[29] Vandemoortele G., Eyckerman S., Gevaert K. (2019). Pick a Tag and Explore the Functions of Your Pet Protein. Trends Biotechnol..

[30] Zhang J., Zhou T., Shan Y., Pan G. (2022). Generation of RYBP FLAG-HA knock-in human embryonic stem cell line through CRISPR/Cas9-mediated homologous recombination. Stem Cell Res..

[31] Zotova A., Pichugin A., Atemasova A., Knyazhanskaya E., Lopatukhina E., Mitkin N., Holmuhamedov E., Gottikh M., Kuprash D., Filatov A. (2019). Isolation of gene-edited cells via knock-in of short glycophosphatidylinositol-anchored epitope tags. Sci. Rep..

[32] Grützkau A., Radbruch A. (2010). Small but mighty: How the MACS-technology based on nanosized superparamagnetic particles has helped to analyze the immune system within the last 20 years. Cytom. A.

[33] Wang G., Yu G., Wang D., Guo S., Shan F. (2017). Comparison of the purity and vitality of natural killer cells with different isolation kits. Exp. Ther. Med..

[34] Rufino-Ramos D., Lule S., Mahjoum S., Ughetto S., Bragg D.C., Pereira de Almeida L., Breakefield X.O., Breynea K. (2022). Using genetically modified extracellular vesicles as a non-invasive strategy to evaluate brain-specific cargo. Biomaterials.

